# Patients with chronic obstructive pulmonary disease treated by the mobile emergency care unit - hospitalization and prognostic factors

**DOI:** 10.1186/2197-425X-3-S1-A393

**Published:** 2015-10-01

**Authors:** KP Lindvig, AC Brøchner, AT Lassen, S Mikkelsen

**Affiliations:** University of Southern Denmark, Odense C, Denmark; Institute of Clinical Research, Mobile Emergency Care Unit, Department of Anaesthesiology and Intensive Care, University of Southern Denmark, Odense C, Denmark; Department of Emergency Medicine Odense University Hospital, Odense C, Denmark

## Introduction

Chronic Obstructive Pulmonary Disease (COPD) is an important and increasing cause of morbidity and mortality worldwide. Patients with COPD suffer from acute exacerbations (AE), which lead to a reduced quality of life, increased risk of mortality, further and longer hospitalization, and increased healthcare costs.

## Objectives

The aims of the study are to describe patients with AE admitted through the Mobile Emergency Care Unit (MECU), to identify prognostic factors and to determine the associated 30-day mortality.

## Methods

The study was performed in the MECU in Odense Denmark, from the 1^st^ of July 2011 - 31^st^ of December 2013. All first-time contact patients (>18years) with COPD treated by the MECU within this period were eligible for the study. AE diagnosis were confirmed by patient record review otherwise the patient was excluded from the study.

## Results

Within the study period of 2 years and 5 months, 438 patients with AE were treated by the MECU, hereof 264 (60.3%) patients were first-time contacts, and thus eligible for inclusion in the study. Furthermore, the MECU had 174 (39.7%) additional patient contacts with AE within the study period. Of the 264 included first-time contacts, 113 (42.8%) were male, and the mean age was 72.4 years. 159 (60.2%) had severely affected breathing upon arrival of the MECU. 7 patients (2.6%) were intubated in the prehospital setting. 262 (99.2%) of all patients with AE in contact with the MECU were admitted to hospital, and only 2 (0.76%) patients were left at scene, this might be due to the fact that only first-time contacts were included in the study. Patients were hospitalized on average 5.3 days (0-48). 22 of 264 (8.3%) patients were transferred to the intensive care unit (ICU) with an average stay of 2.6 days (56.6 hours), of these 22 patients, 21 (95.5%) were mechanically ventilated on average 38.7 hours. 7/22 (31.8%) of patients admitted to ICU were dead within 30 days. The overall 30-day mortality among patients with AE was 12.5% (33/264). In a multivariate Cox regression model, age>80 years HR 0.8 (0.2-3.0) and female sex HR 0.8 (0.4-1.9) were analyzed as prognostic factors of mortality among patients with AE, however were found non-significant at a 95% confidence level.

## Conclusions

We found that among first-time contact patients with AE treated by the MECU, a small proportion of these patients are admitted to ICU to receive mechanical ventilation. However one third of these patients die within 30 days. Despite non-significant levels, data indicate that when admitted to hospital due to AE, being male sex and of younger age worsens the prognosis.

**Figure 1:**
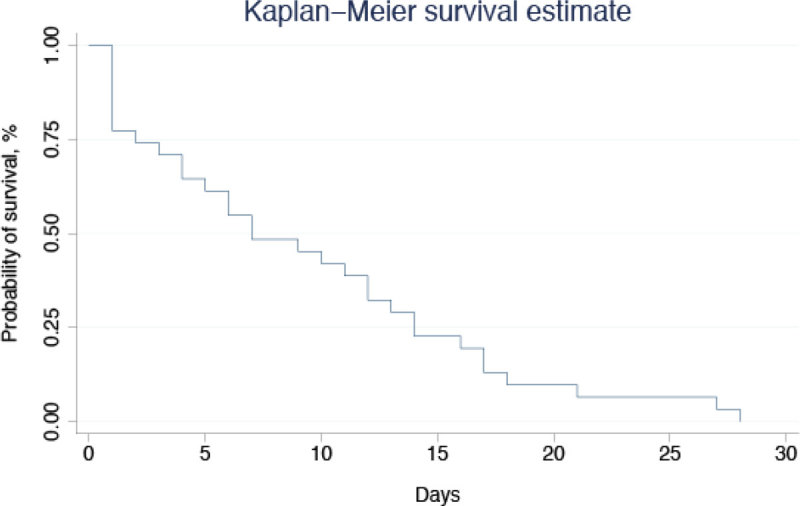
**Kaplan-Meier Survival estimate.**

